# Book Reviews

**Published:** 1814-03

**Authors:** 


### CRITICAL ANALYSIS
### OF RECENT PUBLICATIONS
IN THE
### DIFFERENT BRANCHES OF PHYSIC, SURGERY, AND MEDICAL PHILOSOPHY.

*Des Maladies de la Vessie et du Méat Urinaire, chez les Personnes avancées en Age ; pour servir de réponse aux Questions proposées en* 1807, *par l'Académie Josephine de Médecine et de Chirurgie de Vienne.* Par M. NAUCHE, Médecin de Bienfaisance du 4e Arrondissement, Membre et ancien President de la Société Galvanique, &c. &c. &c. 8vo. Paris, 1810. pp. 252.

THE Josephine Academy of Medicine at Vienna, in the year 1807, proposed as the subject of their prize the following very important questions, viz. "What are the diseases of the bladder and urethra, besides the stone, in old people, which are sooner or later fatal? What are their causes? How are they distinguished from each other, and from the stone; and what is the most appropriate treatment for them?" The present treatise was intended by the author as an answer to these questions, but he was too late to contest the prize, and resolved to be beforehand with the Academy in publishing his work previous to the adjudication of the reward.

If the success of surgeons in treating diseases bore any proportion to the labor bestowed on the investigation of them, or to the number of books which have been written upon them, the sufferers from the affections of the bladder and its appendages would have reason to congratulate themselves on the attention they have attracted. But the diseases which were formerly fatal are unfortunately still so; and the management of those which are curable, is scarcely at all improved. To the number of books on this subject which have *not* advanced our knowledge or means of curing these diseases, the treatise of M. Nauche may be added; although it is, with some exceptions, a very good abridged history of the complaints it treats of, comprehending, however, not only those diseases which are peculiar to old age, but nearly all the various affections of the urethra and bladder.

After some remarks, which might have been spared, such as, that the history of diseases is more attended to and cultivated than the means of cure, that the signs of the presence or absence of calculi are as equivocal and delusive now as they have ever been, &c. the author proceeds ceeds to the consideration of the peculiarities of the bladder in old age.

"La vessie qui éprouve de si grands changemens dans les premières années de la vie, en offre moins dans un âge avancé : son état, à cette époque, diffère peu de celui qu'elle a dans l'âge adulte.

"Cet organe, chez les vieillards, s'arrondit par son sommet, devient plus large transversalement que d'avant en arrière ; il ne s'élève au-dessus du détroit supérieur du bassin que dans son état de plenitude, et ses rapports avec la paroi antérieure de l'abdomen sont moins étendus.

"Il acquiert, lorsqu'il a perdu de son irritabilité, une grande capacité, et devient plus mince. Souvent cette capacité est moindre que dans l'âge adulte, et ses parois sont épaisses et comme racornies.

"A mesure que l'on avance en âge, la vessie perd de son irritabilité; elle devient moins sensible à l'impression de l'urine, et n'en détermine la sortie qu'à de grands intervalles; le jet que le liquide forme est moins long et s'efface presque entiérement.

"L'urètre est moins ferme et a moins de ressort que dans l'adulte; il est plus rempli de sang et parsemé de vaisseaux variqueux.

"L'urine est rendue en plus grande quantité que dans les autres âges: elle est plus visqueuse, plus chargée de matières salines, et spécialement de phosphate de chaux ; on la voit plus fréquemment altérée par les maladies des voies urinaires.

"*Des maladies de la vessie et de l'urètre auxquelles les vieillards sont exposés.*—Les maladies de la vessie, quoique fréquentes à toutes les époques de la vie, attaquent cependant d'une manière plus spéciale les personnes avancées en âge, lesquelles sont moins exposées aux affections de l'urètre que dans un âge moyen. Indépendamment du calcul de la vessie, qui, n'étant pas compris dans le programme de l'Académie, ne sera traité que secondairement dans ce Memoire, les personnes d'une âge avancé sont sujettes à divers accidens resultant des vices de conformation de la vessie et de l'urètre, à des deplacemens de ces parties, à des blessures, à l'inflammation, au spasme, à la paralysie, à des varices, à des fongus, au cancer, à la presence de corps étrangers dans leurs cavités, à l'endurcissement de la prostate, au rétrècissement de l'urètre, etc.

"Ces affections donnent lieu à deux vices dans l'excrétion de l'urine: l'incontinence et la rétention, et par suite à la rupture de la vessie ou de l'urètre, aux dépôts urineux et aux fistules urinaires.

"Parmi ces maladies, il en est de communes à tous les âges, bien décrites par les auteurs, sur lesquelles nous nous ètendrons peu : d'autres, à raison de leur fréquence et de leur ténacité, peuvent être regardées comme propres aux persónnes d'un âge avancé, dont elles sont un des plus grands fléaux, Elles n'ont pour ainsi dire été qu'indiquées par la plupart des auteurs; telles sont l'inflammation lente, le catarrhe chronique, la paralysie de la vessie : nous les considérerons sous tous leurs rapports, et nous tàcherons d'en exposer avec précision les caractères essentiels ainsi que le traitement."

The succeeding chapters, on malformations, herniæ,

 introversion, introversion, and wounds of the bladder, contain little more than selections from the authors who have discussed those subjects, and seem to have no good claim to a place in what professes to be a work on the diseased bladder of old people. The author then treats of inflammation of the three several coats of the bladder. The inflammation of the peritoneal coat, as far as the bladder has that covering, differs in nothing from peritoneal inflammation in general; but the inflammation of the muscular coat is discussed more at large. It is either acute or chronic; it affects either the fundus or the neck of the bladder: but the acute stage, in either case, according to the author, runs its progress in from six to ten days; it is produced, in old people, by the presence of calculi, by catheteric applications, by cantharides taken internally, and, finally, what is always at hand to account for diseases, "la repercussion d'un vice dartreux, psorique, ou gouteux." There is nothing peculiar in the author's plan of treatment, and the symptoms of this species of inflammation are, we think, rather too nicely distinguished from the inflammation of the *mucous* membrane of the bladder, which is the subject of the next chapter. We should, however, make one remark on the use of the catheter in retention of urine as a symptom of this disease: the author directs, that the instrument should be of the smallest and finest kind, that it may pass freely into the neck of the bladder. Now we believe, that, provided there be no impediment in the urethra, generally speaking, the larger the catheter is the more easily it passes, and the less irritation it excites.

The catarrh of the bladder, or inflammation of its mucous membrane, is thus well described:

"Cette maladie se presente dans deux états, dans celui d'aigu et dans celui de chronique.

"Le catarrhe aigu se manifeste par une tension à l'abdomen, par des douleurs à la vessie, qui se propagent jusqu'à l'extrémité du gland, et qui sont accompagnées de difficultés d'uriner, d'envies de satisfaire ce besoin, quelquefois de rétention d'urine complète.

"L'urine, d'abord rouge et trouble, dépose par le refroidissement, le troisième ou le quatrième jour de la maladie, une matière muqueuse, blanchâtre, visqueuse, très-adherente au fond du vase.

"Le pouls est ordinairement accéléré, la langue sèche, la fièvre plus ou moins forte, sur-tout lors de l'invasion de la maladie.

"Après une durée de vingt à trente jours, ce catarrhe se termine ordinairement par resolution; quelquefois il occasionne la mort du malade, lorsqu'il est compliqué de l'inflammation de la membrane musculeuse; souvent il se termine par un engorgement indolent de la membrane muqueuse, qui donne lieu au catarrhe chronique de cette membrane.

"Cette maladie est fréquente chez les personnes avancées en âge: celles celles d'un tempérament phlegmatique y sont plus exposées que les autres. Elle peut être produite par un refroidissement subit, une suppression de transpiration, sur-tout pendant un tems humide et froid, dans les constitutions catarrhales; par une irritation dans la vessie, provenant de la présence d'un calcul ou d'un corps étranger; par des injections de substances trop irritantes dans ce viscère, etc.

"Ce catarrhe est encore dû à la disparition d'une affection dartreuse, psorique et rhumatismale.

"La goutte peut encore l'occasionner. Les narcotiques donnés imprudemment à l'intérieur quand cette maladie occupe les articulations, l'application sur ces dernières, ou sur les ulcères goutteux, de topiques astringens, fortifians ou narcotiques, une disposition du sujet et un premier degré de débilité de la vessie produisent quelquefois le transport de la goute sur cet organe.

"Le catarrhe aigu de la vessie serait peu dangereux par lui-même; mais il l'est souvent à raison de sa complication habituelle avec l'inflammation de la membrane musculeuse de la vessie."

The inflammation of the prostate gland, as distinct from its chronic induration, the author considers as frequently a venereal symptom, and as one that may be subdued by mercurial friction. The symptoms are well described, but we must doubt the accuracy of some of the causes which are assigned, and we must enter our protest against the following mode of treatment: "When we have not been able to subdue the inflammation, and a fluctuation is felt in the tumor formed by the prostate, (query, is this fluctuation ever felt?) we must hasten to open it by plunging a bistoury deep into it from the perinæum."

The inflammation of the urethra, or gonorrhœa, occupies a long chapter; but, as it contains nothing very new or important, and as our readers will readily allow that this is not peculiar to old age, we shall only remark that, in opposition to the opinion of most writers, the author conceives it is no uncommon circumstance for this complaint to produce the true Lues Venerea.

The Hæmaturia is considered as either essential or symptomatic, and the two species are distinguished by the former being an exudation of fluid blood mixed with urine, from the vessels, without any wound; while the latter is an erosion, or wound, of those vessels, and the blood is usually passed without pain, and without the action of the abdominal muscles, that is, without the act of urinating. There is a third species of Hæmaturia, a flow of blood from the kidneys, which is very difficultly distinguished from the former kinds, but is usually attended with more pain in the lumbar region.

We shall not enter into the various causes assigned for this disease, in which the author, in common with most French

French authors, is very fertile. The treatment is often attended with difficulty; the remedies which will arrest the hæmorrhage in some instances, will in others accelerate it. Cold water, and ice, applied to the loins and abdomen, acidulated drinks, lime-water, a recumbent position, gentle laxatives, and spare diet, are the principal means relied on for the cure. When the complaint resists this plan of treatment, more active astringents, in which we have but little faith, are advised; and when the urethra is obstructed with coagulated blood, producing retention of urine, the catheter is recommended, and ablution of the bladder with warm water, or warm water and quarter part of lime-water added to it; but these remedies seem to us of doubtful efficacy.

A long train of symptoms is described as peculiar to spasm of the bladder and urethra; as spasm, without considering these symptoms in the manner of our less discriminating surgeons, as produced by the distension of the bladder. In these the catheter cannot be introduced during the attack of spasm, but is received with facility after the complaint has subsided. We wish the author had enlarged more fully on this important subject, as it appears to us that a great proportion of those complaints which are called stricture, are to be classed under this head.

There is a very good chapter on those affections of the urethra which are produced by varicose vessels in the canal, which is not commonly treated of by authors, but is, we believe, in old people, a very common cause of suffering, and which we shall probably take another opportunity to examine.

---

*An Account of a successful Method of treating Diseases of the Spine; with Observations and Cases in Illustration.* By THOMAS BAYNTON, of Bristol, Author of a Treatise on Ulcers. 8vo. pp. 128. Longman and Co.

The treatment which Mr. Baynton recommends, is entire rest in the horizontal position. "Resting in the horizontal position, is as effectual in *improving circulation*, *favouring the deposition of bone*, *and promoting absorption*, as it is in preventing pressure, and allaying pain." In his early cases, the author conjoined with rest, the use of drains; in his later practice, he has not found them generally, if ever necessary, in the cure of diseases of the spine. In these cases, when a tonic remedy is indicated, he occasionally prescribes muriate of lime, but does not place great confidence in its alleged beneficial effects in scrofula. As rest is the chief means employed in the cure, it is essential that it should be obtained obtained with the greatest ease to the patient, and that excoriation, and other inconveniences consequent upon long confinements in bed, should be avoided. For these purposes the author suggests, that—

"A crib, or narrow bedstead, must be procured, six feet in length, or rather, of a sufficient length to accommodate the patient; two feet one inch in height from the floor of the apartment to the floor of the crib, whereon the mattress is placed; two feet five inches wide, with posts three feet seven inches high, (including castors) to be turned (by a turner) as a common crib.

"It must be provided with a rail floor, instead of sacking; and with side boards to raise up and down; which, when half raised, will resemble the raised flap of a table, and must be supported with sliders, that can be drawn in or out when required; and which, when wholly raised, will furnish sides to the crib, for security, or warmth at night. The castors should be of brass, and of the strongest description.

"This crib is to be fitted with a mattress, from three to four inches thick, that has been French, or double stuffed, with the best horse hair; made two inches shorter, and two inches narrower than the crib, in the clear of the sides, head, and foot-board; for the purpose of affording room for raising the sides, and turning in the bed-clothes. Its width will be sufficient for the accommodation of the patient, though its dimensions will admit it to be drawn through the doorways of the patient's day and night apartments. Its height, when just the mattress is placed upon it, will be sufficient to raise the patient to the level of a common table."

By this means the patient may be readily moved from one apartment to another, and thus have the advantage of changing the atmosphere, and enjoying the pleasure of society.

The author has annexed several cases in support of his practice, and we feel much satisfaction in inserting some of them for the benefit of the public.

"The friends of Miss ——, about 16 or 17 years of age, requested my attendance at the village of Bishport, near Bristol, on the 5th of December 1802.

"On my arrival I was introduced to three young ladies, who were sitting together in a parlour apartment, all apparently in good health, and remarkably cheerful. The parent of the lady who was to become my patient, asked me if I could discover the invalid, and on my replying in the negative, she told me that her daughter, whom she then pointed out, was completely palsied by a disease of the spine, for which sea-bathing, and a mechanical apparatus for the removal of pressure, had been a long time used without the slightest advantage.

"She also informed me that her application to me had been made in consequence of the recommendation of Mr. ——, a gentleman of the the most distinguished eminence in London, for the advantage of whose opinion she had, by the advice of her physician, gone with her daughter to London, and had just returned; having previously thereto procured a new set of machinery, from a person I believe named Jones, by the advice of the gentleman above alluded to.

"On examining the diseased part, I found a considerable projection of two of the dorsal vertebræ, and the contiguous parts in a very tender state.

"I also found that the sensibility of the legs, thighs, and other parts below the diseased, was so completely destroyed as to make it impossible to excite the slighest sensation, by pinching, pricking, or any other means.

"I considered it a duty to apprize the relations of this interesting and very amiable young lady, that much time ought not to be lost in the trial of the new apparatus, if relief were not experienced. It however unfortunately happened, that a period of more than five months was employed in its trial.

"On the 15th of the following May, 1803, I was requested again to see this lady, and to adopt any means which I might consider to be likely to afford a chance of recovery. By this time a remarkable change had taken place. The cheerfulness and appearance of health, which at my first visit had been so astonishingly preserved, as to have occasioned no perceptible difference between her and her healthy companions, were entirely gone. Her general appearance was now so unfavourable, as to occasion me to fear that her life could not be preserved till a trial could be given to the means I intended to have recourse to.

"One great point was however gained by this apparently unfortunate state of circumstances. A determination had been made by the very anxious relations, to adopt, implicitly, any means that might be recommended.

"She was immediately placed on an unyielding mattress without a pillow; setons were inserted on each side of the diseased vertebræ, and instructions were given to observe, with the utmost care, that the body should never be raised from the horizontal position, or moved for any other than natural purposes, or the dressing of the setons. A suitable regimen, and medicines adapted to the varying circumstances of the case, were exhibited. The general health soon became improved; but it did not happen till the eleventh month that any sensation was experienced in the palsied parts. About that time a tingling was felt in the legs; and from that period the recovery proceeded so rapidly, that at the end of the fifteenth month she could walk without inconvenience, and by the end of the eighteenth month could run, leap, and dance, as well as she ever could in her life.

"The protruded processes of the vertebræ continued to project, in consequence of the occurrence of anchylosis previously to the adoption of recumbency.

"I have had frequent opportunities of hearing from this lady and her friends, that no tendency to relapse, nor any inconvenience, has been been experienced since the completion of her cure, a period of nearly ten years."

"On the 17th of April, 1811, I was consulted by the friends of Miss ——, aged 15, on account of a disease of the spine, for which setons had been recommended. Upon examination, a slight lateral curvature was observed, and on pressing the processes, it was discovered by the tenderness, that some of the dorsal vertebræ were in an inflamed state. Absolute rest, in the manner described, was therefore recommended; and as the general appearance indicated that a tonic remedy was necessary, the muriate of lime was ordered; no material affection of the parts below the diseased had occurred, nor had the general health been much injured.

"I did not see this lady a second time. She was removed to the country, and the directions observed there. A perfect cure was obtained in six or seven months, but of the exact time I am not certain."

"Master Wm. Castle, son of Thomas Castle, esq. of Portland-square, Bristol, when about a year old, was observed to have lost his health, and it was discovered that he could not move his legs as well as he had been accustomed to do. On examining the spine I discovered that four of the lower dorsal vertebræ were in a diseased and protruded state, and that great pain was occasioned by moderate compression, either of the bones or contiguous soft parts. Absolute rest on a hair mattress was strictly enjoined; the muriate of lime, in appropriate doses, directed, and the constant care of a confidential servant ensured. The advantages resulting from those means were soon apparent; they continued progressively to increase till the cure was accomplished. At the end of the tenth month he was allowed to roll and play on a carpet; in a short time after he got on his feet without assistance, and has continued in perfect health from that time to the present. The curve is entirely removed."

From the perusal of Mr. Baynton's judicious remarks, and reflecting upon the success which he has happily obtained, we have no doubt that the practice will prove efficacious in many cases. He has, however, candidly mentioned some instances of a disease in the hip-joint, as well as a disease of the spine, in which the means he recommended failed. Considering the nature of the disease, we are not surprised at this circumstance; indeed we think Mr. Baynton has been very fortunate to have experienced so few disappointments in the cure of a malady which often resists the utmost efforts of art. From the short view we have given of the author's publication, our readers may try his practice for themselves; but we would also advise them to obtain the work itself, which contains much useful information in a small compass.

*Anatomy of the Heart, Cranium, and Brain, adapted to the Purposes of the Medical and Surgical Practitioner; to which is added, in Notes, Observations on the Laws of Life and Sensation.* By ALEXANDER RAMSAY, M.D. Lecturer on Anatomy and Physiology in Edinburgh. 4to. Fasciculus I. pp. 66. Second edition, much enlarged. Longman and Co. London; Constable and Co. Edinburgh. 1813.

*A Series of Plates of the Heart, Cranium, and Brain, in imitation of Dissections* (to accompany the preceding). 4to. Second edition. By ALEXANDER RAMSAY, M.D. &c. Longman and Co. 1813.

As a teacher, we remember Dr. Ramsay was particularly curious in his demonstration of the brain, and successful in displaying its admirable conformation. It gives us pleasure, therefore, on this occasion to renew our acquaintance with him, and extend the knowledge of his work amongst professional readers. Although the present number forms the first fasciculus of a complete system of anatomy, it may be regarded as a distinct work upon the subject of which it treats. The author has even dived into metaphysics, and will probably obtain more readers out of the profession than in it. He is a decided anti-materialist, and has taken great pains to distinguish those operations of mind which depend upon material organs, and those principles or qualities of the soul which are independent of them. This effort of the author produced the following observation from Sir Joseph Banks: "I give you abundant thanks for your most laudable attempts to destroy the baneful system of materialism, and to explain the hitherto incomprehensible mixture of mortality and immortality of which we are composed."

The work is materially assisted by the plates, which are executed in the style of dissection; and by an ingenious contrivance of the author's, the various complicated parts of the brain can be separated from each other, or be made to assume the appearance of an united whole. This has been attempted formerly, but Dr. Ramsay's contrivance is better adapted than any we have yet seen.

The letter-press embraces two objects—A minute anatomical description with references (which are also given in the text), where the thread of demonstration is not interrupted by any physiological inductions. 2dly, The physiology of each subject in the text is given in notes, immediately beneath the anatomical description. Thus the speculative philosopher may investigate the wonderful doctrines of the animal and intellectual economy of man, without the fatigue of making making himself familiar with those anatomical minutiæ, so essential to the medical character—who again may avoid perplexing his understanding, or encumbering his memory, with the author's metaphysical opinions.

We shall not dwell upon the anatomical part of the work, as it is the production of a man whose lectures on the subject are well known, and his present performance is sanctioned by the names of several eminent physicians, anatomists, and teachers. Some of Dr. Ramsay's doctrines, however, are curious, and somewhat novel: we shall introduce a portion of them to our readers' notice.

The author commences with the heart and arteries, and to their operations assigns the universal phenomena exhibited, not only in the growth and progressive stages of the animal being of man, in health and disease, but also those ideas or operations of mind which are gradually developed. Even the ideas of man, in a material state of existence, he observes, owe their origin to corporeal organs.

"We discover these promoting the growth and attachments of the child. They build up organs which give being to the functions and feelings of puberty. The genius and varied stages of maturity succeed. These same organs decay. New and corresponding modifications fill their place. Man is intended to advance from one period of perfection to another, as an intelligent and moral agent; and when the heart and arteries fail to actuate him as an animal, their physical offices, after mid-life, leave him at leisure to contemplate, more minutely, the nature and intention of his immortal being, through the same progressive medium of natural organs which affected the previous periods of life. The brain seems continually actuated through the medium of these primary agents; the state of which as physically induces solid and exalted ideas in old age, unexcited by vigour, sexual organs, and in possession of wisdom; as in youth we discover lively fancy, or ambitious froth, flowing from the presence of vigour and sexual organs, in the absence of experience. I must be understood to consider these objects as an anatomist, so far as mortal and changing organs have an influence in promoting the phenomena connected with an immortal essence.

"The heart and its vessels seem to derive their powers as acting organs from their muscular economy. From the vitality of these, on which the varied modifications of action depend, all physical phenomena seem to derive their origin, whether we contemplate life, growth, health, disease, the varied talents of men, or the phenomena of decrepitude and dissolution.

"The vitality of muscles would seem dependent on some superadded principle, which is continually applied to an inherent state of organization, which presents a capability or capacity, admitting of excitement by the various appropriate stimuli connected with animal being; for the vital organs, although continuing the same, instantly expire in the absence of the atmospheric and other fundamental

 agents; tagents; on the other hand, when the organs have accomplished their various stages, stimuli are applied in vain. Mortality seems to be a necessary termination of living organized bodies; the inherent capability or capacity for being excited, seems to suffer diminution by every pulsation the heart and vessels perform. The same aliment, atmosphere, solar and other influences, therefore, which excite living action, likewise promote the process of death; and those active external powers which administer to the living, precipitate the valetudinary and the dead to decomposition, again to form new and endless compounds. Life itself may really be deemed a living decomposition, of which the various stages of existence are periods, and death its termination, as an organized whole.

"Muscular matter, from its influence on the nervous system, appears the acting agent on which the result of sensations depend. If heat, cold, light, or darkness, communicate certain actions to the vessels, which are taken notice of by the soul, through the medium of the nerves and brain, as objects of reflection—by whatever means these actions take place, must not the same mental conclusion follow? Mankind are naturally apt to confound ideas connected with sensation, which are operations of *soul,* with *soul itself.* Nothing seems a stronger illustration of the immutable nature of *soul,* than the mutability of *ideas,* or operations of *mind,* so far as they are connected with sensation. In this transitory state of existence, the *immortal soul* is informed through the medium of *mortal organs,* so far as sensation is concerned;—a single rash action, or inordinate thought, may, in an instant, subvert the economy of these organs, and then the profound mind shall exhibit the distraction and incoherence of the maniac. We startle at these awful results of morbid states of the heart and arteries, forgetting that the possession of understanding is only a consequence of another modification of the same organic operation. Could we expect, where a wise God is concerned, that one species of action should occasion its appropriate intellectual effect, and that another should fail in its corresponding consequence? We look with calmness and expectation at that state of organs, the action of which induces pleasure and a sane mind; but we startle when inordinate action induces its corresponding ideas. But both states prove to us the influence of corporeal organs (continually obnoxious to change) on the unchangeable intellectual principle which takes cognizance of these changes; and these phenomena are the true results of corporeal function in the collected mind, as well as the unhinged understanding, both of which exhibit those intellectual phenomena which correspond with corporeal health or disease of this fabric, which composes the material instrument of the soul. We may therefore denominate muscular economy the physical basis of sensation, and such reflections of intellect as flow from such material operation. Hence the laws of *Religion* are the rules of *health* and *correct ideas.* The *healthful* and *sober* man, sees and feels things as they are, because the muscles of his heart and arteries are only excited when objects are present to excite them. But *disease* and *intemperance* induce a state of system, when the *legitimate* operations of muscles are *suspended,* and of their own accord they assume those actions tions (on which sensations depend) in the absence of exciting causes. On such occasions, the phantoms of clouds and darkness often occur in clear sunshine—light and flashes of fire are apparent when we are enveloped in the real gloom of night—various colours float before the eye of the valetudinary—nauseous tastes, ungrateful odours, confused sounds, and teazing feelings, distract and mislead the victim of morbid action, which have no existence but in the convulsions of imbecile muscular matter, which often fails, on the other hand, to be actuated by natural appropriate stimuli. Pleasant sensations are rarely experienced by a morbid state; pleasure is that action, depending on a state of system prepared for and dependent on the reception of physical stimuli. The notion of morbid strength, therefore, in the paroxysms of febrile and other maladies, seems very erroneous; as these actions are really the convulsive and involuntary struggles of unhinged nature, which never take place in health or strength. We discover in *articulo mortis,* that the individual has no power to effectuate voluntary exertions; but the muscles of the limbs of their own accord, contrary to will, contract with violence—the penis is often suddenly erected—the muscles of the abdomen, rectum, and vesiculæ seminales expel their contents, &c. These seem not the consequences of strength, but the results of expiring muscularity, which never takes place in a state of strength, but in debility. The same observation holds respecting the morbid action of the heart and arteries. The heart and arteries of a healthy man contract gently on the application of their contents. Suppose this man exposed to excessive fatigue, endemic miasmata, or any debilitating cause; the imbecility induced, occasions the same blood to promote the convulsive throb of fever. The discreet application of cold effusion shall in a short time restore lost energy, equipoise takes place, and convalescence of the system is assumed. Do these circumstances denote morbid strength —or morbid action from induced debility? Phlebotomy is only another means of attempting the same end. Contraction is the physical action of muscle, while relaxation is its forced state. When the transverse fracture of the patella occurs, the rectus contracts, but never is relaxed of its own accord. On this simple law would I be induced to explain the varied phenomena of the animal economy. Without any exertion on our part, the sphincter ani observes its physical contractility, till forced by air or feces to yield to superior power; and we can demonstrate the difference between physical and induced contraction, by the stimulus of the mind, or any foreign body affecting the anus, as by these applications it is thrown into a violent and convulsive state of contraction. In the levator palpebræ too, we discover the distinction between physical and induced contraction. When fatigue has overwhelmed the powers of nature, the levator palpebræ, which has been continually in an induced state of contraction by mental influence, is overpowered by the superior contractile physical power of the sphincter oculi,—the eye continues shut till nature has been restored to energy by the suspension of mental influence during sleep. When organic energy is consummated, mental cognizance again occasions the contraction of the levator palpebræ, and the sphincter returns to a relaxed state.

In

In a note, p. 14, the author conveys a hint to philosophers, to investigate anatomical structure and the leading causes which influence living matter, before they assign reasons for the variety in human figure, genius, and maladies. Note, p. 21, affords some suggestions on apoplexy and obesity; p. 45, observations on idiotism, hunger, &c. The following remarks on the latter subject are curious: "This morbid hunger seems a very universal state of feeble subjects, and would appear to depend on the action of the gastric fluid on the languid state of the stomach, of which healthful hunger would appear a species, though more easily allayed. Does death-hunger, as it is vulgarly termed, support this opinion? In the fevers of America, wherever patients expressed sudden and violent desire for food, they never lived; they often died with the bread in their mouth, mingled with the blood which flowed from their gums and nostrils. On the contrary, while active fever is assumed, the irritable state of the stomach rejects food, I observed the most salutary effects take place frequently (when the stomach and brain exhibited the most morbid symptoms), from applying to the forehead a number of layers of a towel, wrung out of cold water, and this cold kept up. In such cases, cold affusion was inadmissible; the stomach revolted at every application; the gentle stimulus of the cold was diffused, and the stomach was allayed in its irritability. Vapour bath had a similar effect."

In note, p. 49, is a comparison between the brain of the human and of the brute species; and at p. 51, some ingenious remarks occur on sensation, the finer senses, and certain intellectual phenomena. Dr. Ramsay regards the nerves as "agents intervening between the material world and the soul."

"Wherever we view the animal machine, the actions communicated to the heart or arteries seems ultimately impressed on the nerve, and thence to the sensorium commune. The office of muscular fibre seems action. We discover the heart pulsating, though torn from its cavity and nervous appendages; and the whole mass of muscles of an animal, after the head is separated, shall tremble for a time by the stimulus of the atmospheric fluid, or the application of a pointed instrument. The office of the nerves seems that of reception of actions. All sensations seem modifications of touch. When you apply the tip of your finger to an object, that object affects the living vessels, the action of which is impressed on the nerve, and thus communicated to the brain. Since arteries are formed of a congeries of muscular annuli, and muscles contract on application of stimuli, (nerves being composed of medullary and cineritious matter, interwoven with living vessels) these communicate the action to the nerves, which correspond to the impingence received by arteries, from material or intellectual influence. The shock of a thought becomes comes occasionally as fatal as the flash of lightning. The same observation applies to the other senses, which are touched by their appropriate objects, as the eye, &c. These convey or do not convey sensation, as bodies adapted to excite them are applied. The same applications and actions, however, which occasion sensation, may have being, and no sensation take place; which seems to afford a clear demonstration between mere life and action, and intellectual operation; and proves that, however material organs may render us acquainted with a material world, these organs, as well as the objects impressed on them through the medium of their structure and offices, are known to us only through the medium of the soul. When we investigate correctly these physical and intellectual phenomena, their true and distinguishing characters seem so unequivocal, that we must be surprised that so many great men should, by listening to preconceived opinions, embrace contrary and absurd extremes, by neglecting to look at nature, and failing to revere religion. Motion and ideas are both realities; they are each distinct consequences of matter and intellect. Pain and pleasure of body are as much intellectual operations, dependent on connection with material organs, as compunction or self-approbation are purely intellectual results, flowing from the laws of our Maker, written on our mind. Whatever is discovered by human nature, physically or intellectually, becomes so from the presence and attention of soul. If I agitate the point of your finger, you complain of titillation; but when you are sound asleep, though the same irritation may be applied, and yet be unfelt, the same exciting cause, as well as its corresponding physical arterial action, has taken place. Does this lead us to a key, by which may be opened the mysterious fact, of the distinction between body and mind? The person who reads this is all over alive; his wonderful fabric is a thrilling being, suspended between life and death; of this fabric, however, at this moment, he has no cognizance, if in health. The mind has no cognizance of absolute healthy action, till excitement takes place. Deep and mortal wounds communicate little or no sensation, though inflicted even on the hemispheres of the brain, till morbid action occasions cognizance: a proof of sensation having being where nerves are not concerned. If you read this subject with attention, your right hand, as well as every other part of your corporeal system, is unknown to you, till I brought you to recognize this circumstance, that you are possessed of organs of which you are not conscious: but you had complete cognizance of the subject before us, and that alone. The actions therefore necessary to sensation seem to depend on a certain state of motion in the vessels, without which there is no cognizance. Mere life and health is a consequence of arterial action, unconnected with cognizance. Sensation of pleasure seems a gentle agitation of the vessels and nerves, and pain an inordinate convulsion of the same organs, both of which are taken notice of by the soul. Disease, therefore, is a continual cognizance of morbid action; and even excess of lawful action predisposes to disease."

For a further elucidation of this subject, see the note in p. 53 and 54. This note concludes with hints and reflections upon upon madness. At page 57 we meet with a direction for dissecting the eye, which we believe was first introduced by Dr. Ramsay in his school at Edinburgh.

Note, p. 59, gives a new view of the distribution of the nerves supplying the muscles of the eye, the author contending that diversity of nerves is necessary to superinduce similar actions in dissimilar muscles—as agents supplied by similar nerves assume with difficulty those motions which are dissimilar. For the illustration of this doctrine, see note, p. 59 and 60.

We have now presented our readers a slight sketch of Dr. Ramsay's first fasciculus: its conclusion, page 6[illegible], contains more piety than is usually met with in books upon anatomy, and we think the work especially adapted to interest the taste and gratify the curiosity of *serious* readers. The student, indeed, may, through its medium, become acquainted with anatomy, though he may not have time to enter upon those abstruse speculations which occupy the greater portion of the notes. We understand the work is highly and illustriously patronized;* that the succeeding fasciculi are nearly ready, and will present, in the plates, a system of dissections that may be studied without handling the knife, or offending the senses.

It has been shrewdly observed that there is no royal road to science; but in the work before us, we have a royal road to anatomy, and a sharp cut at materialism.

* The second number will be dedicated by permission to the Duke of Kent.

